# Progressive Intracranial Arteriovenous Fistula Over Forty Years: A Case Report

**DOI:** 10.7759/cureus.88348

**Published:** 2025-07-20

**Authors:** Jaime Lois F Opinion

**Affiliations:** 1 Neurosurgery, Vicente Sotto Memorial Medical Center, Cebu, PHL

**Keywords:** digital subtraction angiography, hemorrhage, intracranial dural arteriovenous fistula, temporal artery ligation, venous ectasia

## Abstract

This report highlights the rare progression of a dural arteriovenous fistula (dAVF) over four decades, evolving from an initially benign presentation to aggressive hemorrhagic features. A 58-year-old male presented with uncontrollable scalp bleeding secondary to a pulsating mass on the right temporal area. Diagnostic cerebral angiography revealed a complex right transverse sinus dAVF with multiple arterial feeders, primarily from the bilateral superficial temporal arteries (STA) and occipital arteries via the external carotid arteries (ECA). Internal carotid artery (ICA) injections failed to adequately opacify due to advanced atherosclerotic changes. No cortical venous reflux or venous ectasia was identified; instead, a serpiginous network of ECA feeders was observed, likely due to collateral development following a prior ligation in 1979. Clinically, the patient’s only presenting complaint was persistent scalp hemorrhage from the vascular mass. He exhibited no neurological deficits preoperatively, and no steal phenomena or other typical dAVF-related symptoms were observed. The hemorrhage, initially manifesting as anemia, was managed conservatively with transfusions before definitive treatment. Surgical ligation of the ECA feeders was performed via an open technique. As this did not involve an endovascular embolization, no immediate postoperative angiogram was obtained. The patient showed significant clinical improvement and was discharged in a stable condition without any neurological deficits. This report underscores the importance of patient compliance, long-term follow-up, and timely neurosurgical intervention in managing progressive dAVFs, particularly those evolving over decades with atypical presentations.

## Introduction

Intracranial dural arteriovenous fistulas (dAVFs) are acquired, pathologic connections between dural arteries and either dural venous sinuses, meningeal veins, or cortical veins [1]. These lesions account for approximately 10-15% of all intracranial arteriovenous malformations (AVMs) [2]. Most dAVFs manifest in individuals in their fifth to sixth decades and commonly involve the transverse, sigmoid, or cavernous sinuses [1]. This report describes a rare case of a dAVF first diagnosed at 17 years of age in 1978, during an era when diagnostic modalities were limited to carotid angiography via direct cervical puncture [3]. Initial treatment typically involved proximal ligation of feeding arteries, a method that achieved complete occlusion in only 45% of cases [4].

Although the patient underwent right external carotid artery (ECA) ligation, no detailed diagnostic imaging was available at the time to map all feeding vessels. During a recent cerebral angiography, the right ECA remained patent, suggesting that the initial treatment had been incomplete. Rather than true recurrence, this case reflects a slow progression of the original fistula, with progressive recruitment of extracranial arterial feeders bilaterally from the ECA system over nearly 40 years. The pathophysiologic progression of dAVFs is believed to be driven by chronic venous hypertension, angiogenesis, and recruitment of collateral vessels, particularly in incompletely treated or inadequately monitored cases. In this patient, serpiginous angiogenic growth likely developed due to insufficient vessel occlusion and the natural course of vascular remodeling over time.

Progression from a benign, asymptomatic state to a hemorrhagic and clinically aggressive form remains exceedingly rare. A series by Hetts et al. [5] reported such conversion in only 3.2% of patients (19 out of 579). While dAVFs are often categorized by their venous drainage pattern and clinical symptoms, ranging from pulsatile tinnitus to ophthalmoplegia or hemorrhage, this patient’s presentation was atypical, with no neurologic deficits despite an enlarging, actively bleeding scalp mass. This report thus underscores the critical importance of long-term surveillance, especially in cases with incomplete initial treatment and limited early diagnostics. It also emphasizes the evolving role of modern neuroimaging and surgical management in addressing progressive or atypical dAVF cases.

This article was previously presented as an oral presentation at the 17th Asian Australasian Congress of Neurological Surgeons (AACNS 2024), the 2024 Annual Meeting of the Taiwan Neurosurgical Society, and the 2024 Interim Meeting of the Taiwan Academy of Neurosurgery on November 17, 2024, in Kaohsiung, Taiwan. Written informed consent was obtained from the patient, and institutional review board approval was secured for the publication of this case report.

## Case presentation

A 58-year-old male presented to the Emergency Department of Vicente Sotto Memorial Medical Center with active scalp bleeding originating from a pulsatile mass in the right temporal region. On physical examination, the mass was noted to be large, firm, and highly vascularized. The patient was hemodynamically stable but anemic. Initial stabilization was followed by a detailed clinical evaluation and diagnostic cerebral catheter angiography under local anesthesia and sedation, utilizing the Seldinger technique. Although axial or coronal CT or MRI images could not be retrieved due to a change in the institution’s imaging service provider in 2022, official radiologic reports were available. These indicated no evidence of intracranial hemorrhage or infarction, aside from a localized hematoma noted at the left temporoparietal area.

The patient reported a history of a scalp mass first noted in 1978, which had reportedly bled profusely and had been managed by ligation of the right ECA at that time. No follow-up, imaging, or further treatment had been performed for nearly 40 years. There had been no interval diagnostic surveillance. Over the years, the patient had observed a gradually enlarging, pulsatile mass in the right temporo-parietal region. The current episode of hemorrhage had been triggered by mechanical irritation while combing his hair, resulting in a sudden and profuse gush of blood, prompting emergency consultation. Notably, diagnostic cerebral angiography had not been available at the institution during the earlier years; it only became routinely accessible in the late 2010s.

**Figure 1:**
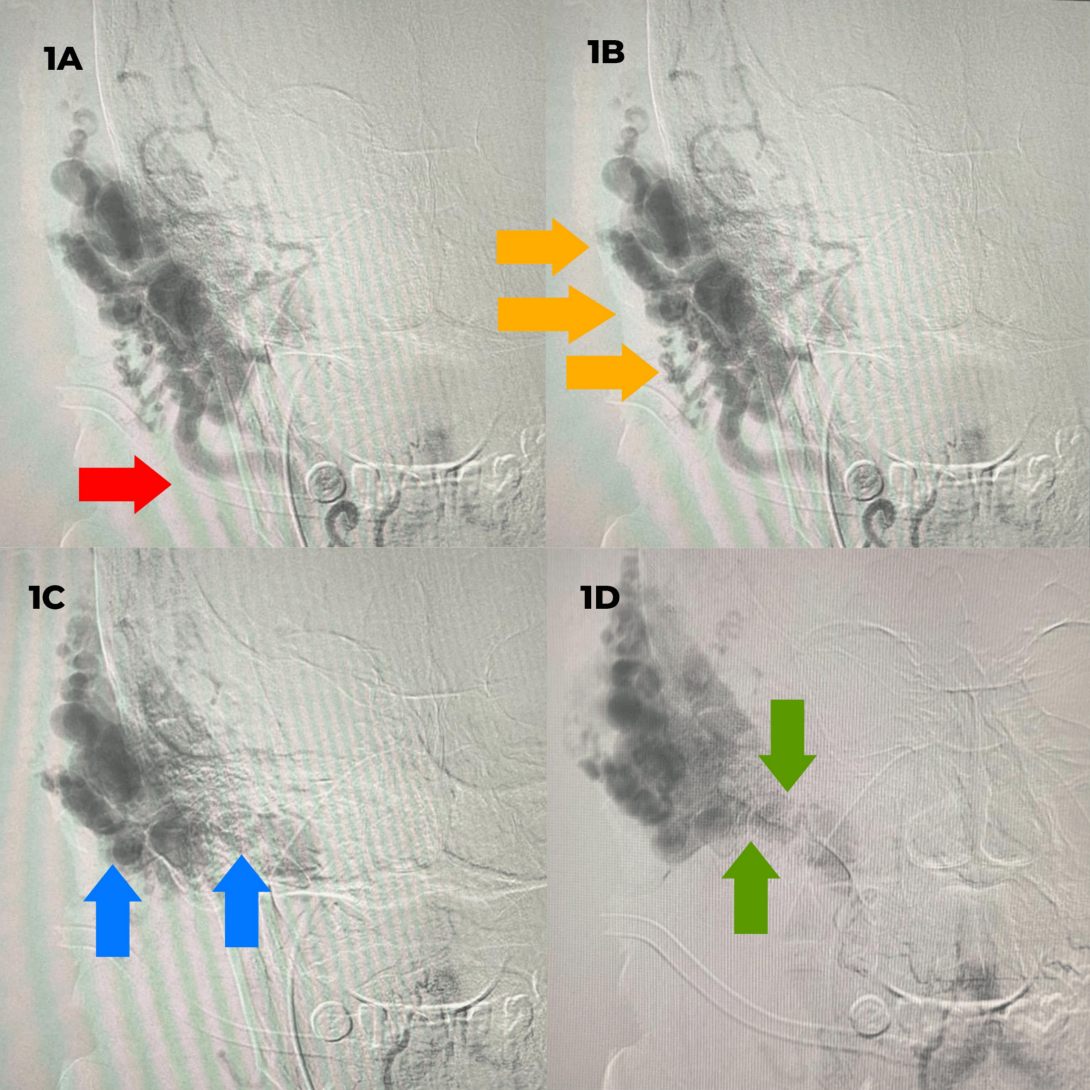
ECA injection showing a complex dilated arterial feeders forming a complex scalp mass and a dilated transverse sinus dural arteriovenous fistula 1A: Main ECA trunk (red arrow). 1B: Complex scalp mass at the right temporoparietal area (yellow arrow). 1C: Same ejection showing delayed arterial phases draining into the transverse dural arteriovenous fistula (blue arrow). 1D: Transverse sinus dural arteriovenous fistula (green arrow) ECA: external carotid artery

Cerebral angiography revealed a diffuse dAVF involving the right transverse-sigmoid sinus. On the venous phase of the right ECA angiogram, there was prominent opacification of the fistulous network with arterial supply arising from the superficial temporal artery (STA) and branches of the right occipital artery, confirming the diffuse nature of the transverse-sigmoid dAVF. Figure 1 illustrates delayed ECA injection showing main tributaries from the right STA (red arrows) and the fistulous connection at the transverse-sigmoid junction (green arrow).

**Figure 2:**
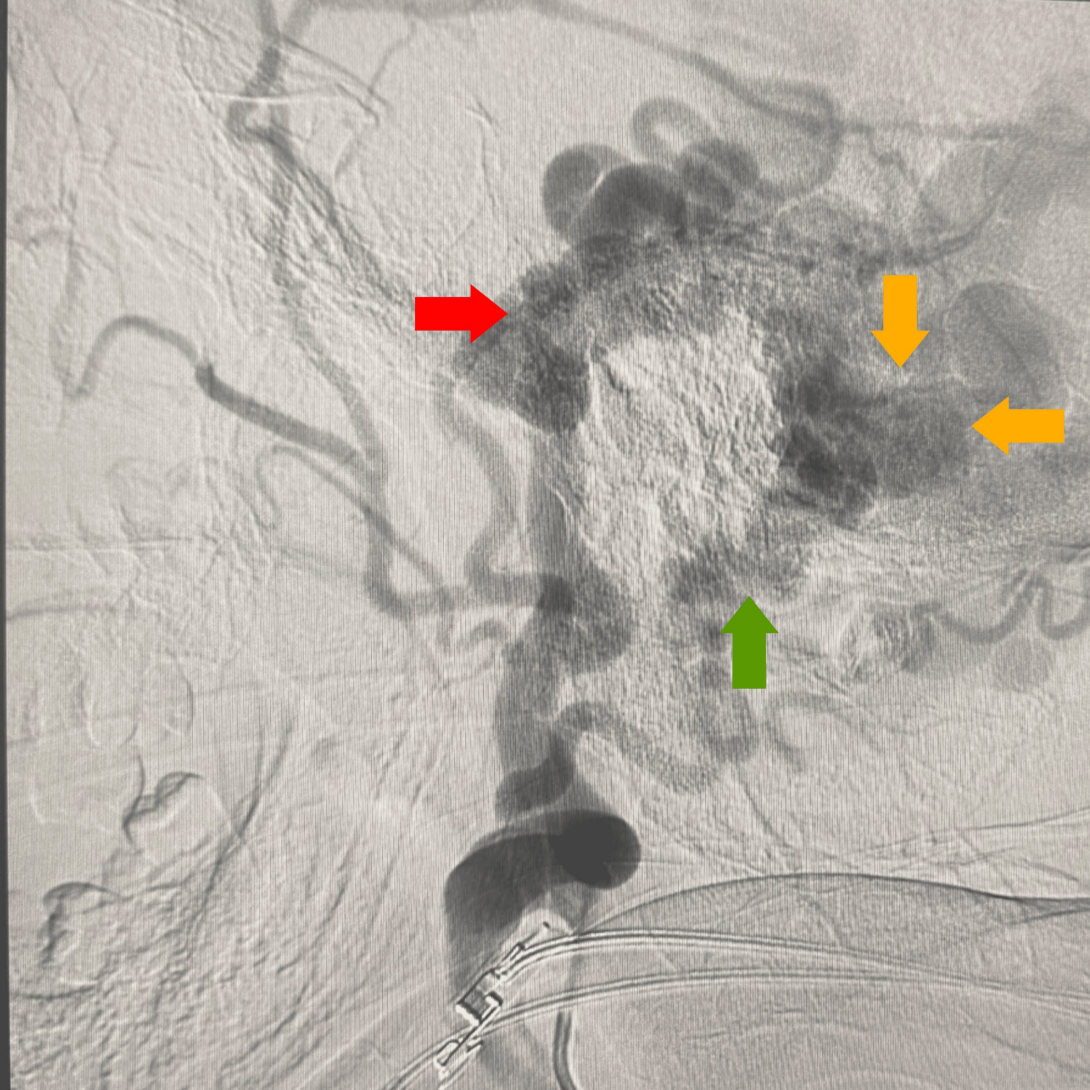
Right lateral ECA injection showing dilated feeders forming a complex scalp mass The right superficial temporal artery (red arrow) and the right occipital artery (green arrow), which are the main tributaries of the transverse sinus dural arteriovenous fistula (yellow arrow) ECA: external carotid artery

Based on the angiographic characteristics, the lesion was classified as Cognard Type I, indicating antegrade drainage into a dural sinus without cortical venous reflux. Further injections of the right ECA highlighted a complex vascular mass within the scalp, receiving multiple arterial feeders from both the STA and occipital artery, correlating with the visible pulsatile lesion. Figure 2 displays the right lateral ECA view showing a dilated vascular scalp mass (red arrows).

**Figure 3:**
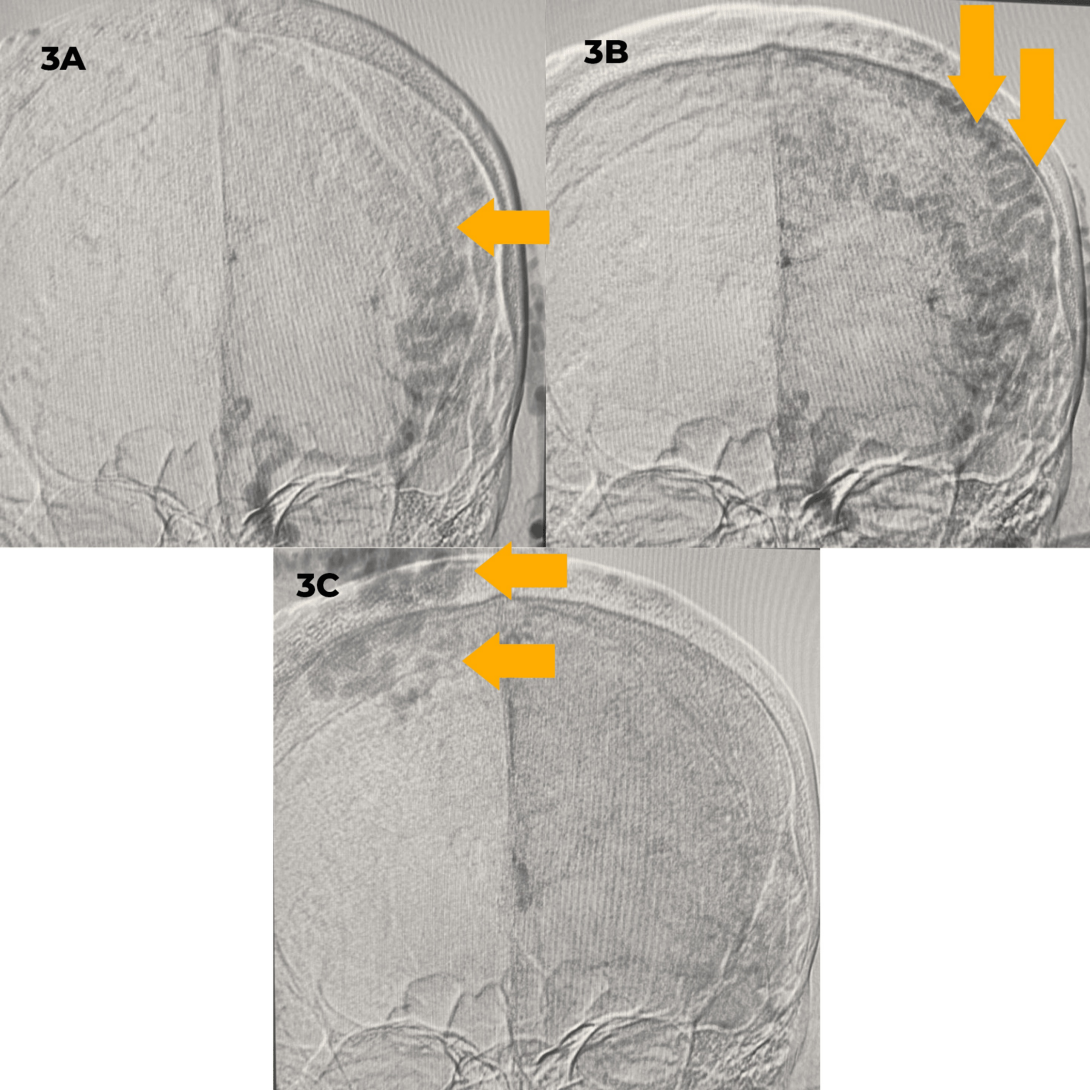
Coronal CCA injections showing multiple dilated left superficial temporal artery feeders going to the transverse sinus dural arteriovenous fistula (yellow arrows) 3A: The image shows a common left carotid injection, showing multiple dilated feeding arteries from the left superficial temporal artery. 3B: The image shows dilated multiple superficial temporal artery feeders about to cross the midline into the right transverse sinus dural arteriovenous fistula. 3C: The image shows the final path of the multiple superficial temporal arteries draining into the right transverse dural arteriovenous fistula CCA: common carotid artery

Selective distal ECA injections provided additional delineation of the fistula, revealing extensive arterial contributions not only from the STA but also from the middle meningeal artery, reinforcing the complexity and high-flow characteristics of the lesion (Figure 3).

To quantify contralateral supply, left common carotid artery angiography was performed. Early arterial phase images demonstrated cross-filling from the left common carotid artery, showing multiple dilated feeders from the STA crossing midline, going to the fistula.

Figure 3, obtained during an early right ECA injection, demonstrated the complex scalp mass with clearly visualized STA and occipital artery tributaries, emphasizing the high-flow characteristics of the lesion. These multimodal imaging findings confirmed the bilateral, high-flow nature of the dAVF, with contributions from both extracranial and vertebral systems. In addition to the extensive arterial feeders, angiographic findings also included no venous ectasia and evidence of intracranial atherosclerosis, raising concerns for potential rupture or further hemorrhagic complications.

Given the diffuse, bilateral arterial supply and the acute presentation with scalp hemorrhage, the patient underwent bilateral ligation of the ECA feeders. Intraoperatively, scalp tributaries from both hemispheres were traced. A vertical slit incision was made to expose the STA and occipital artery bilaterally. Ligation was performed as close as possible to the approximated site of the transverse-sigmoid junction, the presumed location of the arteriovenous shunt, to ensure proximal control of the feeders near the shunt point. The procedure successfully reduced shunting through the fistula, resulting in cessation of scalp bleeding. The patient’s anemia was corrected with transfusion therapy, and no neurological deficits were observed during the postoperative course. He was subsequently discharged in stable condition.

## Discussion

This case report demonstrates the rare natural history and progressive transformation of an initially benign dAVF into a complex, symptomatic, and hemorrhagic lesion over four decades. While dAVFs are often acquired lesions, their behavior ranges from benign to highly aggressive depending on their angioarchitecture and venous drainage patterns [6]. In this patient, the lesion’s long-term progression may be attributed to several factors, including recanalization of previously occluded vessels, neoangiogenesis induced by chronic venous hypertension, and the upregulation of vascular endothelial growth factor (VEGF) pathways [7]. These pathophysiological mechanisms are well-documented in progressive dAVF cases and are associated with worsening cortical venous reflux and higher hemorrhagic risk.

Using the Cognard classification, the lesion in its current form corresponds to Type I, which indicates antegrade flow into a dural venous sinus without cortical venous reflux. However, the extensive arterial recruitment from bilateral superficial temporal and occipital, suggests an increase in shunt volume and flow demand over time. Although no cortical venous reflux was noted on angiography, the presence of serpiginous vascular channels and scalp venous dilation suggests evolving venous hypertension. It is plausible that this lesion began as a Cognard Type I and progressively increased in flow dynamics, potentially nearing transition to a higher-risk subtype had it remained untreated.

The differential diagnosis for a bleeding scalp mass includes scalp AVM, cirsoid aneurysm (a type of pseudoaneurysm), and dAVF. In this case, digital subtraction angiography (DSA) ruled out a scalp AVM due to the absence of an intervening nidus. A cirsoid aneurysm was also excluded due to the lack of characteristic dilated, tortuous vessels isolated to the subcutaneous layer. The angiographic features supported a diagnosis of a diffuse transverse-sigmoid sinus dAVF. The classic work by Newton and Cronqvist emphasized the ability of dural arterial feeders to adapt and recruit new supply routes over time, especially from external carotid branches such as the superficial temporal and occipital arteries [3]. This mirrors our angiographic findings, where bilateral superficial and occipital feeders contributed to the extensive fistulous network. Notably, the bilateral and vertebral artery contributions, and the 40-year silent progression of the lesion, represent one of the longest reported natural histories for a dAVF transitioning from asymptomatic to hemorrhagic.

The transition of dAVFs from benign to aggressive forms has been well-characterized. Hetts et al. identified cortical venous reflux as a key risk factor for neurologic deterioration and hemorrhage [5]. Bulters et al. further reinforced that lesions with cortical venous drainage warrant aggressive surveillance and early intervention, given their elevated risk profiles [8]. In this case, the lesion’s delayed presentation with hemorrhagic complications likely stemmed from long-standing venous hypertension and insufficient follow-up.

Despite advances in imaging, DSA remains the gold standard for diagnosing and evaluating intracranial dAVFs [6]. While endovascular embolization is often the preferred treatment in well-resourced settings, the decision to proceed with open surgical ligation in this case was influenced by both anatomical feasibility and economic considerations. In low- and middle-income countries (LMICs) like the Philippines, access to endovascular therapy is often limited due to cost. A complete embolization involving both STAs, both occipital arteries, and vertebral feeders would have cost an estimated PHP 600,000-700,000, which would be borne by the patient or family. In contrast, open surgical ligation required only standard surgical materials such as sutures and skin staplers, significantly reducing the financial burden.

This report also underscores the continued relevance of traditional neurosurgical techniques, such as vessel exposure and targeted ligation near the fistulous point, as an effective and viable option in resource-limited settings. This case draws attention to systemic healthcare challenges, particularly in the Philippines. Limited access to specialized care, fragmented referral pathways, and socioeconomic factors often delay patient follow-up. As this report illustrates, lesions left untreated or undertreated may silently evolve into life-threatening conditions. The Department of Health’s National Objectives for Health 2017-2022 emphasizes the need for improved continuity of care and equitable access to neurosurgical services [9].

In summary, this report contributes new insights into the extended natural progression of dAVFs, demonstrates unique bilateral ECA contributions, and reinforces the value of surgical adaptability in LMICs. It illustrates the importance of long-term monitoring, patient education, and timely treatment in preventing complications associated with dAVFs. It also underscores the need for health systems in resource-limited settings to develop mechanisms that support continuity of care and early intervention.

## Conclusions

Intracranial dAVFs can progress over time and may transform from benign to life-threatening conditions. This report highlights the importance of timely diagnosis, long-term monitoring, patient education, and surgical intervention, especially in settings where modern interventional options may be unavailable. Strengthening continuity of care may prevent complications in similar cases. While endovascular embolization remains the preferred approach in many settings, this case reinforces the importance of preserving microvascular surgical skills among neurosurgeons. In resource-limited settings, open surgical ligation remains a cost-effective and life-saving alternative. Clinical training programs should continue to equip neurosurgeons with a range of treatment modalities to ensure comprehensive care across diverse healthcare settings.
